# Case report: Paternal uniparental disomy on chromosome 7 and homozygous *SUGCT* mutation in a fetus with overweight after birth

**DOI:** 10.3389/fgene.2023.1272028

**Published:** 2023-10-17

**Authors:** Xiufen Bu, Xu Li, Can Peng, Hongyu Li, Shihao Zhou, Zesen Zhu, Jun He, Siyuan Linpeng

**Affiliations:** ^1^ Hunan Provincial Key Laboratory of Regional Hereditary Birth Defects Prevention and Control, Changsha Hospital for Maternal and Child Health Care Affiliated to Hunan Normal University, Changsha, China; ^2^ Department of Physiology, Changsha Health Vocational College, Changsha, China; ^3^ Technical Support Center, Zhejiang Biosan Biochemical Technologies Co., Ltd., Hangzhou, China

**Keywords:** paternal uniparental disomy, prenatal diagnosis, *SUGCT*, overweight, invasive prenatal diagnosis, case report

## Abstract

**Background:** Paternal uniparental disomy (UPD) of chromosome 7 is extremely rare, and only a few postnatal cases have been reported. The effects on growth were discordant in these cases, and the relevance of paternal UPD(7) to growth caused by imprinting remains questionable.

**Case presentation:** Here, we report a prenatal case that underwent invasive prenatal diagnosis due to the high risk of Down’s syndrome and failed noninvasive prenatal screening. The fetus had a normal karyotype and no apparent copy number variation. Homozygous copy-neutral regions on chromosome 7 were identified using a single nucleotide polymorphism (SNP) array; the data for the parent–child trios showed that the fetus carried the whole paternal isodisomy of chromosome 7. Whole exome and Sanger sequencing revealed a homozygous frameshift mutation in *SUGCT* at 7p14.1, from the heterozygous carrier father, with no contribution from the mother. The parents decided to continue with the pregnancy after genetic counseling, and the neonate had normal physical findings at birth and showed overweight after birth during a long-term intensive follow-up.

**Conclusion:** We report the first prenatal case who carried paternal UPD(7) and homozygous *SUGCT* mutation with an overweight phenotype after birth. The overweight may be caused by paternal UPD(7) or homozygous frameshift mutation of *SUGCT*, or both of them, but it is unclear which contributes more.

## 1 Introduction

Uniparental disomy (UPD) refers to the presence of one or more chromosome pair(s) derived from only one parent in a disomic cell with a balanced karyotype (Liehr, 2014). It can be paternally or maternally derived; it may affect the whole genome, a chromosome, or a part of it. Heterodisomy (hetUPD) is the inheritance of two homologous but genetically different chromosomes from one parent. Inheritance of two identical copies of a single homolog is termed isodisomy (isoUPD). There are fewer isoUPD cases than hetUPD cases, and the number of paternal-origin UPD cases is one-third that of maternal-origin UPD cases (Benn, 2021). In maternal UPD, isoUPD is less common than hetUPD, while in paternal UPD, the frequencies of both conditions are almost equal (Benn, 2021). The incidence of single whole-chromosome UPDs is non-random, and chromosomal size is not related to UPD formation. The results from four million population screens indicated that the most frequent UPDs involve chromosomes 1, 4, 16, 21, 22, and X, with whole-chromosome UPD occurring in 1/2000 births and double UPDs (two chromosomes at the same time) occurring at a rate of approximately 1 in 50,000 births (Nakka et al., 2019). The incidence of UPD was substantial higher in individuals with abnormal phenotypes. (Yauy et al., 2020; Scuffins et al., 2021).

An abnormal UPD phenotype can result from the unmasking of a recessive disease caused by a pathogenic variant carried by only one parent or from aberrant imprinting. Currently, maternal-origin UPD involves chromosomes 7, 11, 14, 15, and 20, whereas paternal-origin UPD involves chromosomes 6, 11, 14, 15, and 20, which cause imprinting abnormalities (Del Gaudio et al., 2020). Genomic imprinting influences growth, behavior, and viability (Benn, 2021). Growth disturbances are the most common manifestation; paternal UPDs often enhance growth, whereas many maternal UPDs cause growth restrictions (Reik and Walter, 2001). Maternal UPD(7) results in Silver-Russell syndrome (SRS), a growth-restricted syndrome. It is characterized by prenatal and postnatal growth retardation, relative macrocephaly, body asymmetry, feeding difficulties, and a prominent forehead (Azzi et al., 2015). In contrast, the clinical manifestations of paternal UPD(7) remain unclear; only ten postnatal cases with normal karyotypes have been reported (Liehr, 2023). This includes two patients with no available phenotype. Therefore, the relevance of imprinting disorders caused by paternal UPD(7) to the overgrowth or overweight phenotype remains unknown, and more evidence is needed. Here, we report the first prenatal case of paternal isoUPD(7) leading to a homozygous frameshift mutation in *SUGCT*, identified using multiple diagnostic techniques. The fetus showed normal ultrasonographic features throughout the gestation period. The parents decided to continue the pregnancy after genetic counseling, and the neonate showed overweight after birth in a long-term, intensive follow-up.

## 2 Case presentation

**FIGURE 1:**
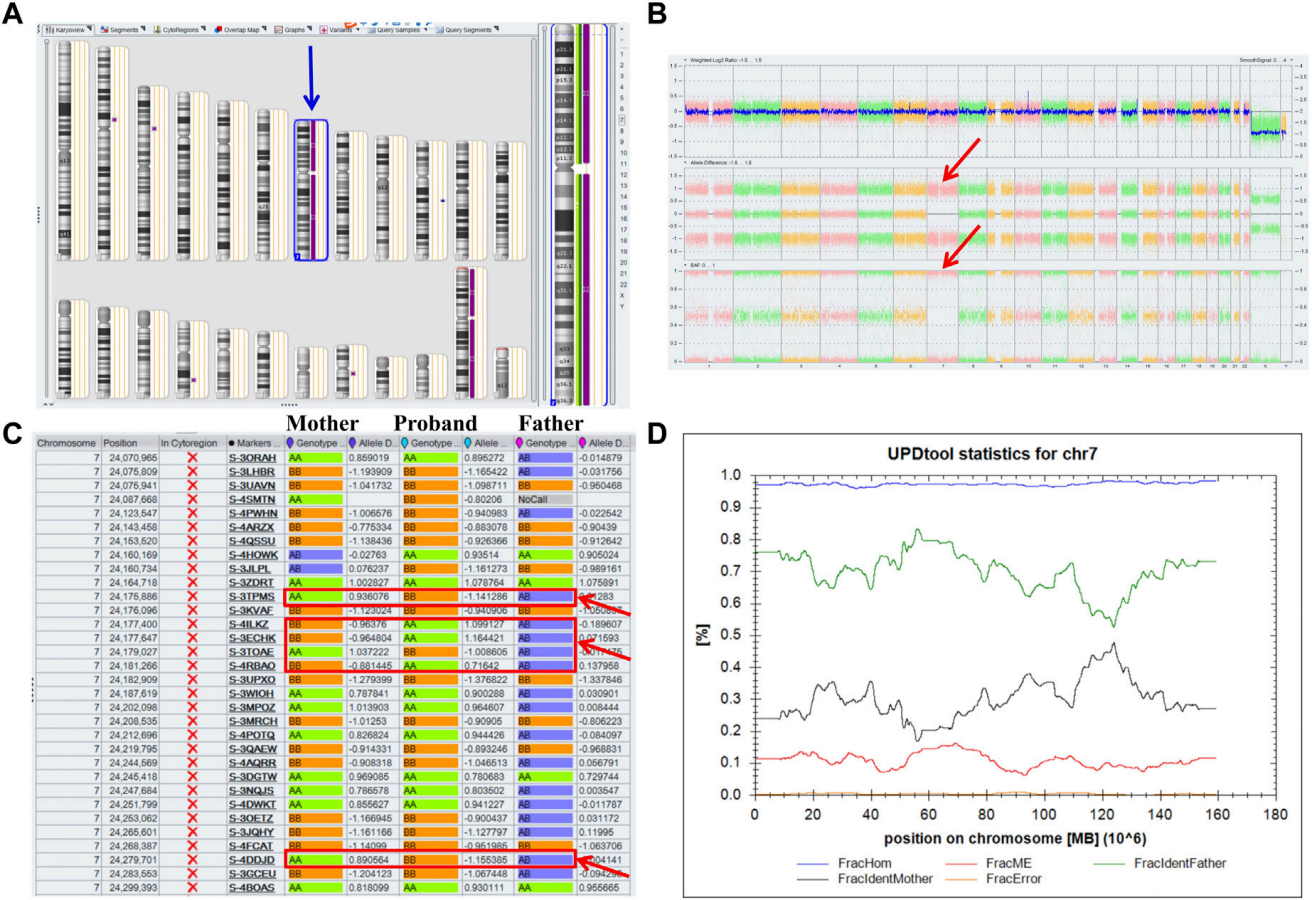
Whole paternal isodisomy (isoUPD) of chromosome 7. **(A)** ChAS revealed a complete regions of homozygosity (ROH) across the whole chromosome (purple rectangle, blue arrow). **(B)** A whole chromosome view clearly shows the copy-neutral ROH on chromosome 7 in the proband (red arrow). **(C)** ChAS software directly indicates that the UPD originated from his father after comparing the genotyping results between the proband and his parents (red arrows). **(D)** Classification of UPD using the UPDtool showed the proband was whole paternal isoUPD. FracHom (blue line) is the fraction of homozygous SNPs, FracME (red line) is the fraction of mendelian error SNPs, FracldentFather (green line) is the fraction of SNPs where the genotype is identical to the father, FracldentMother (black line) is the fraction of SNPs where the genotype is identical to the mother, and FracError (yellow line) is the fraction of errors. The UPDtool is available at: (http://www.unituebingen.de/uni/thk/de/f-genomik-software.html).

**FIGURE 2:**
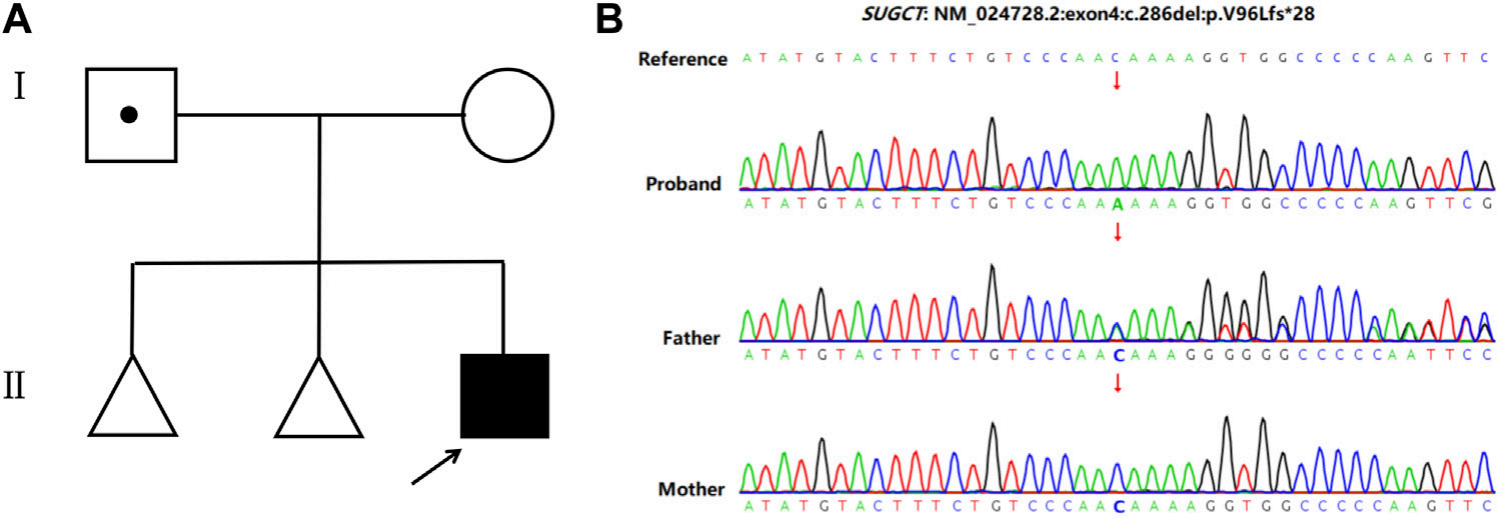
Pedigree of the family and the Sanger sequencing data. **(A)** Family pedigree. The arrows indicate the proband. **(B)** Sanger sequencing showed a homozygous frameshift mutation on *SUGCT* (c.286delC) in the proband, while the father is heterozygous and his mother is normal.

**FIGURE 3:**
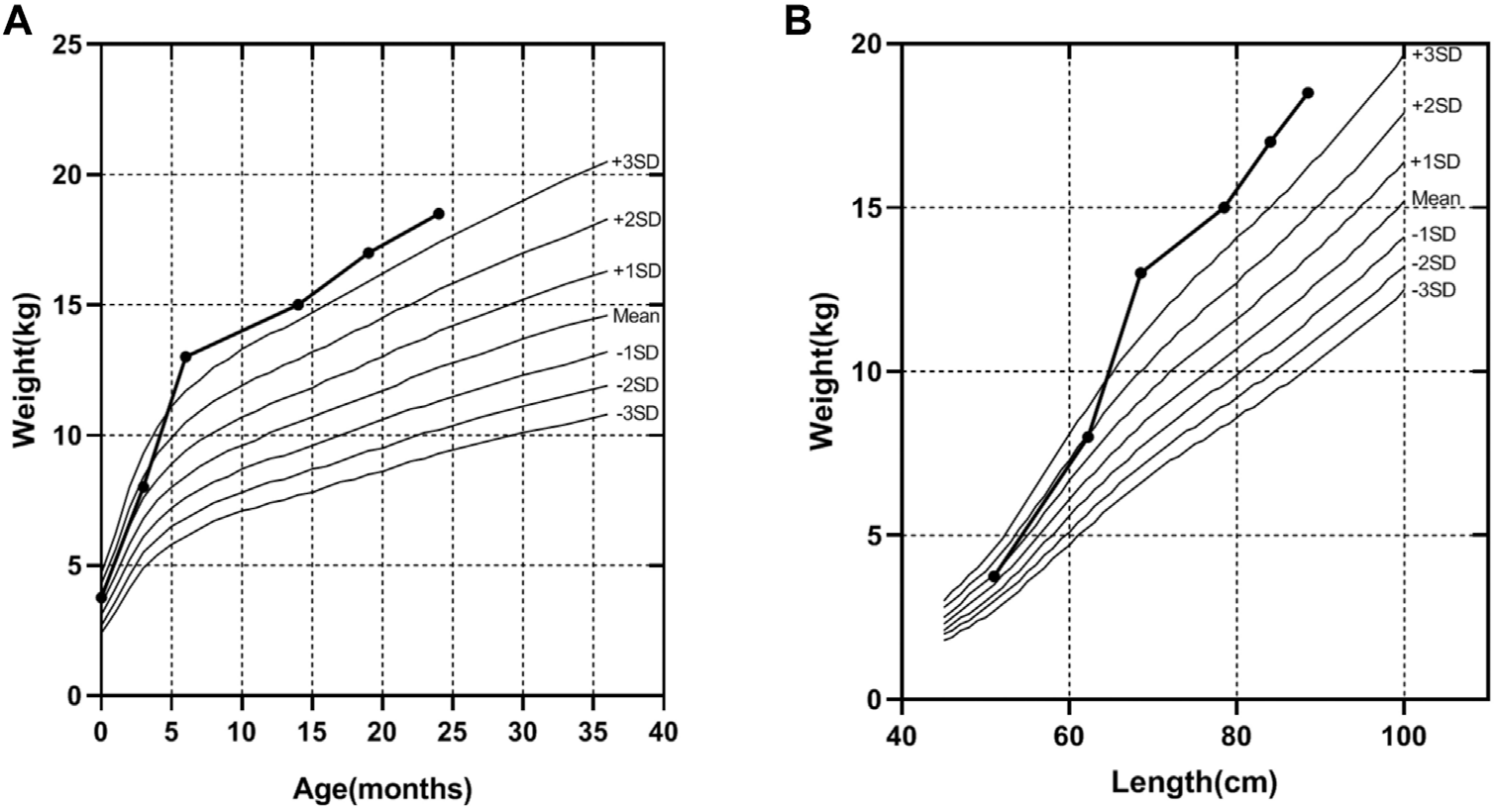
The weight curve of the proband. **(A)** The trend of increasing weight with age of the proband. **(B)** The trend of increasing weight with length of the proband, according to the industry standard of the People’s Republic of China (WS/T 423-2022), from birth to age 24 months.

A 32-year-old healthy woman (gravida 3, biochemical pregnancy 2, para 0) underwent amniocentesis at 21 weeks of gestation because of an adverse pregnancy history, a high risk of Down’s syndrome in early pregnancy (T21 1:150), and two failed non-invasive prenatal screenings (NIPS). G-banding karyotype analysis (320 bands) was performed on the cultured amniocytes. Whole-genome single nucleotide polymorphism (SNP) arrays, whole exome sequencing (WES), and Sanger sequencing of uncultured amniocytes were performed. Karyotype analysis of cultured amniocytes revealed a normal karyotype of 46,XY. Evaluating uncultured amniocytes using an Affymetrix CytoScan 750 K SNP array did not detect pathogenic copy number variants and revealed regions of homozygosity on chromosome 7 (Figures 1A, B). The SNP microarray data of parent-child trios showed that the fetus had complete paternal isoUPD(7), as observed using the Chromosome Analysis Suite (ChAS) software (Figure 1C) and UPDtool (Figure 1D). After genetic counseling, we performed WES on the uncultured amniocytes. The Novaseq6000 platform (Illumina, San Diego, United States), with the 150 bp pair-end sequencing mode was used to sequence the genomic DNA of the family. Sequencing reads were aligned to the human reference genome (hg38/GRCh38) using the Burrows-Wheeler Aligner tool. A homozygous frameshift variant (c.286del:p.V96Lfs*28) of *SUGCT* in exon four was detected on chromosome 7p14.1. We verified this result using Sanger sequencing; the mutation was inherited only from the father, who was heterozygous for this mutation (Figure 2). The parents decided to continue with the pregnancy after genetic counseling, and the fetus presented no structural deformity during the whole pregnancy; the indicators were consistent with the gestational age when measured using ultrasound. At 39 weeks and 2 days of gestation, a male infant was delivered naturally and had normal physical findings, with an Apgar score of 9 at 1 min after birth. The baby underwent physical examination six times from birth to 24 months of age. The length and weight were 51 cm and 3.75 kg at birth, 62.2 cm and 8.0 kg at 3 months, 68.5 cm and 13.0 kg at 6 months, 78.5 cm and 15.0 kg at 14 months, 84.0 cm and 17.0 kg at 19 months, 88.5 cm and 18.5 kg at 24 months, respectively. The length and head circumference (data not shown) were within the normal range while weight was over 3SD from 6 months of age (Figure 3). The family history was unremarkable, and the parents did not have a history of overweight in childhood. Now, aged 2 years, the cognitive, motor and language development of the baby is within average levels. In addition, we evaluated 74 inherited metabolic indicators (including glutaric acid) in blood of the newborn using tandem mass spectrometry; all indicators were within the reference range of our laboratory. At 24 months, urine organic acids were detected using gas chromatography-mass spectrometry, and glutaric acid levels were high (69.47 mmol/mol creatinine, normally less than 8.44).

## 3 Discussion

UPD is a copy-neutral chromosomal variation; however, approximately 65% of the comprehensively-studied UPD cases present with a normal karyotype (Liehr, 2014). This could be attributed to an initial chromosomal imbalance, that is, later corrected to a normal karyotype. Initial meiotic segregation error is a common event that contributes to UPD, and the incidence of meiotic nondisjunction increases with advanced maternal age, independent of paternal age (Nakka et al., 2019). Non-disjunction results in the germ cell harboring disomy or nullisomy, instead of being haploid. In the case of whole chromosomal UPD, there are three primary mechanisms of self-correction (Kotzot, 2008; Conlin et al., 2010; Liehr, 2010): 1) trisomic rescue, in which a normal oocyte (sperm) is combined with a diploid sperm (oocyte) and the supernumerary maternal (paternal) chromosomes in the trisomic zygote are eliminated to restore the normal chromosomal number; 2) monosomic rescue, where the fertilization is between a normal sperm (oocyte) and a nullisomic oocyte (sperm), and subsequent the paternally (maternally)-derived chromosome is amplified; and 3) gamete complementation, where fertilization occurs between a disomic sperm (oocyte) and a nullisomic oocyte (sperm). Trisomic rescue is the main mechanism involved in at least 19% of the reported UPD cases (Liehr, 2014). However, isoUPD has a high correlation with paternal UPD, which in many cases may be due to monosomy rescue (Papenhausen et al., 2011). In this case, both parents were young (mother, 32 years; father, 33 years). Meiotic segregation errors are more likely to occur in the oocytes of women; therefore, paternal isoUPD(7) most likely arises from an anucleate oocyte fertilized by a normal sperm that subsequently duplicates its paternally-derived chromosome.

UPD is closely associated with imprinting disorders. Imprinting is an epigenetic process that results in one allele being silenced, with only one remaining active. In the case of the exclusive presence of paternal or maternal imprinted allele(s), a corresponding syndrome could appear (Prawitt and Haff, 2020). For chromosome 7, maternal UPD(7) results in SRS and is present in 7%–10% of cases (Saal et al., 1993). Several clinical features are characteristic of SRS; the most common is slow growth before and after birth. Babies with this condition have a low birth weight and often fail to grow or gain weight at the expected rate (Eggermann, 2010). There are two separate regions on chromosome 7 (7p11.2-p13 and 7q31-qter) with imprinted genes involved in growth disorders (Butler, 2020). *MEST*, *PEG10*, *CPA4*, *COPG2*, and two imprinted non-coding RNAs (*MESTIT1* and *COPG2IT1*) show a pattern of paternal expression, and they are involved in human growth stimulation. *GRB10* is involved in human growth suppression and is expressed from paternal and maternal alleles in a highly tissue- and isoform-specific manner (Blagitko et al., 2000). It is possible that maternal UPD(7) manifests characteristics of SRS and growth retardation in the presence of two functional maternal copies instead of a normal copy and/or lack of paternally-expressed growth promoter genes (e.g., *MEST*/*PEG10*) (Butler, 2020).

The clinical manifestations of maternal UPD(7) are definite, whereas those of paternal UPD(7) remain unclear because only 13 cases of paternal UPD(7) have been reported (Table 1). Among the complete collection of known paternal UPD(7) cases, there were three with an abnormal karyotype and 10 with the normal karyotype including two individuals without phenotypic information in a methylation profiling study (Table 1; Case 4–5). Among the 3 cases of paternal UPD(7) with an abnormal karyotype, 2 had growth retardation associated with maternal isoUPD(7q) (Table 1; Case 1–2) and 1 died 2 h after birth without detailed growth-related information (Table 1; Case 3). Among these, eight cases had a normal karyotype with available phenotypes, five had cystic fibrosis caused by recessive mutation in *CFTR* on 7q31.2, including four patients with normal growth (Table 1; Case 6–9), and one patient with postnatal overweight (Table 1; Case 10). In the remaining three cases, one patient had congenital chloride diarrhea caused by the recessive mutation in *SLC26A3* on 7q31.1 with normal growth (Table 1; Case 11), one patient was a 5-year-old Japanese boy with an unexplained overgrowth phenotype (Table 1; Case 12), and one had double UPDs; the baby boy exhibited features indicative of Beckwith-Wiedemann syndrome (BWS) spectrum with overgrowth (Table 1; Case 13). In summary, paternal UPD(7) did not affect normal growth in five cases, while three reports suggested paternal UPD(7) have an effect on growth.

**TABLE 1:** Summary of cases with paternal UPD(7).

Case number	Gender	Age of UPD diagnosis	Karyotype	Other genetic abnormalities	Clinical symptoms	Growth
Case 1 Eggerding et al. (1994)	F	6 months	46,XX,i(7) (p10),i(7) (q10)	maternal isoUPD(7q) and paternal isoUPD(7p)	growth retardation, SRS like	growth-restricted
Case 2 Kotzot et al. (2001)	M	12 years	46,XY,i(7) (p10),i(7) (q10)	maternal isoUPD(7q) and paternal isoUPD(7p)	growth retardation, SRS like	growth-restricted
Case 3 Simpson et al. (2007)	M	NB	45,XY, psu dic(7; 7) (p22; p22)	7p telomeric microdeletion and mutation of *FAM20C* in 7p22.3	lethal osteosclerotic bone dysplasia (died 2 h after birth)	NA
Case 4、Case 5 Joshi et al. (2016)	NA	NA	NA	NA	NA	NA
Case 6 Pan et al. (1998); Bartoloni et al. (2002)	M	6 months	N	mutation of *CFTR* in 7q31.2 and *DNAH11* in 7p21	CF, primary ciliary dyskinesia, situs inversus	N
Case 7 Le Caignec et al. (2007)	F	3 years	N	mutation of *CFTR* in 7q31.2	CF	N
Case 8 Feuk et al. (2006)(case 17)	M	postnatal	N	mutation of *CFTR* in 7q31.2	CF, mild language delay	N
Case 9 Goh et al. (2007)	M	33 years	N	mutation of *CFTR* in 7q31.2	CF, congenital bilateral absence of vas deferens	N
Case 10 Fares et al. (2006)	M	2 years	N	mutation of *CFTR* in 7q31.2	CF, delayed development, overweight	overweight
Case 11 Höglund et al. (1994)	F	23 years	N	mutation of *SLC26A3* in 7q31.1	congenital chloride diarrhea, sensorineural hearing loss	N
Case 12 Nakamura et al. (2018)	M	5 years	N	no disease causing gene identified	overgrowth, mitral regurgitation	overgrowth
Case 13 Berland et al. (2021)	M	NB	N	UPD(15)pat and UPD(7)pat	Beckwith–Wiedemann syndrome spectrum	overgrowth
Present case	M	prenatal	N	mutation of *SUGCT* in 7p14.1	overweight	overweight

M, male; F, female; y, years; mo, months; NB, newborn; NA, not available; N, normal; isoUPD, isodisomy; CF, cystic fibrosis; SRS, Silver-Russell syndrome.

The potential genes of chromosome 7 involved in promoting growth are *GRB10*, *PEG10*, and *MEST*. In the cases of overgrowth described above, the boy (Table 1; Case 13) with double UPDs (double paternal isoUPD(7) and isoUPD(15)) showed features of BWS spectrum with overgrowth (height was +2.4 SD and weight +4.0 SD at 10 months of age), placentomegaly, hyperinsulinism, enlarged viscera, hemangiomas, and earlobe creases (Berland et al., 2021). DNA, RNA, and methylation analyses showed that several genes in the gene sets associated with growth were upregulated, and *PEG10* (7q21.3) was the only paternally-expressed gene on these chromosomes that was upregulated more than twice (6 fold). High levels of *PEG10* could exert growth-promoting effects. Hypomethylation of paternal *GRB10*, *PEG10*, and *MEST* was detected in another 5-year-old Japanese boy (Table 1; Case 12) with paternal UPD(7) and overgrowth (height> 3SD, weight> 2SD), indicating that *GRB10*, *PEG10*, and *MEST* could present a dose-superimposed effect to promote growth (Nakamura et al., 2018). In our case, the baby was overweight (>3SD) after birth from 6 months old. Abnormal methylation of the imprinted growth-related gene(s) on paternal chromosome 7 could be involved in this phenotype. However, the influence of imprinting is not limited to individual regulatory elements, but can extend across entire chromosomal domains (Joshi et al., 2016). This may explain why our case, as well as the another (Table 1; Case 10), only showed an increase in weight.

*SUGCT* encodes glutarate-CoA transferase with 434-amino acid. The c.286del:p.V96Lfs*28 variant in *SUGCT* would change the open reading frame of the gene and lead a premature stop codon at amino acid position 124. Loss-of-function mutations in the *SUGCT* gene is associated with glutaric aciduria III (GA3). GA3 is caused by glutarate-CoA transferase deficiency, which decreases the conversion of free glutaric acid to glutaryl-CoA and the isolated accumulation of glutaric acid (Marlaire et al., 2014). This matches the genotype, as the glutaric acid level in our patient was 8.2× higher than the normal upper limit in urine organic acid analysis. GA3 is generally considered a likely “non-disease”, as only few cases with GA3 have been described, and these individuals show asymptomatic or have no single consistent clinical presentation evident. The reported phenotypes have gastrointestinal disturbances, cyclic vomiting, abnormality of brain, *etc.*, but no overweight/obesity (Waters et al., 2018; Dorum et al., 2020; Demir et al., 2023). However, the knockout of *SUGCT* in mouse leads to gut microbiota dysbiosis, metabolic changes, body fat accumulation, and an obesity-related phenotype (Niska-Blakie et al., 2020). Therefore, there is currently insufficient evidence to define whether GA3 causes overweight/obesity.

In conclusion, this is the first prenatal case of paternal isoUPD(7) and *SUGCT* mutation with overweight after birth. The overweight may be caused by paternal UPD(7) or homozygous frameshift mutation of *SUGCT*, or maybe both, but it is unclear which contributes more. Further studies are needed to determine the degree of methylation in the imprinted region or function of *SUGCT* for identifying the cause of overweight.

## Data Availability

The datasets for this article are not publicly available due to concerns regarding participant/patient anonymity. Requests to access the datasets should be directed to the corresponding author.

## References

[B1] Azzi S. Salem J. Thibaud N. Chantot-Bastaraud S. Lieber E. Netchine I. (2015). A prospective study validating a clinical scoring system and demonstrating phenotypical-genotypical correlations in Silver-Russell syndrome. J. Med. Genet. 52, 446–453. 10.1136/jmedgenet-2014-102979 25951829 PMC4501172

[B2] Bartoloni L. Blouin J. L. Pan Y. Gehrig C. Maiti A. K. Scamuffa N. (2002). Mutations in the DNAH11 (axonemal heavy chain dynein type 11) gene cause one form of situs inversus totalis and most likely primary ciliary dyskinesia. Proc. Natl. Acad. Sci. U. S. A. 99, 10282–10286. 10.1073/pnas.152337699 12142464 PMC124905

[B3] Benn P. (2021). Uniparental disomy: origin, frequency, and clinical significance. Prenat. Diagn 41, 564–572. 10.1002/pd.5837 33179335

[B4] Berland S. Rustad C. F. Bentsen M. H. L. Wollen E. J. Turowski G. Johansson S. (2021). Double paternal uniparental isodisomy 7 and 15 presenting with Beckwith-Wiedemann spectrum features. Cold Spring Harb. Mol. Case Stud. 7, a006113. 10.1101/mcs.a006113 34615670 PMC8751407

[B5] Blagitko N. Mergenthaler S. Schulz U. Wollmann H. A. Craigen W. Eggermann T. (2000). Human GRB10 is imprinted and expressed from the paternal and maternal allele in a highly tissue- and isoform-specific fashion. Hum. Mol. Genet. 9, 1587–1595. 10.1093/hmg/9.11.1587 10861285

[B6] Butler M. G. (2020). Imprinting disorders in humans: a review. Curr. Opin. Pediatr. 32, 719–729. 10.1097/MOP.0000000000000965 33148967 PMC8791075

[B7] Conlin L. K. Thiel B. D. Bonnemann C. G. Medne L. Ernst L. M. Zackai E. H. (2010). Mechanisms of mosaicism, chimerism and uniparental disomy identified by single nucleotide polymorphism array analysis. Hum. Mol. Genet. 19, 1263–1275. 10.1093/hmg/ddq003 20053666 PMC3146011

[B8] Copyright (2023). Copyright © 1993-2023. Seattle: University of Washington, Seattle. GeneReviews is a registered trademark of the University of Washington.

[B9] Del Gaudio D. Shinawi M. Astbury C. Tayeh M. K. Deak K. L. Raca G. (2020). Diagnostic testing for uniparental disomy: a points to consider statement from the American College of Medical Genetics and Genomics (ACMG). Genet. Med. 22, 1133–1141. 10.1038/s41436-020-0782-9 32296163

[B10] Demir E. Doğulu N. Tuna Kırsaçlıoğlu C. Topçu V. Eminoglu F. T. Kuloğlu Z. (2023). A rare contiguous gene deletion leading to trichothiodystrophy type 4 and glutaric aciduria type 3. Mol. Syndromol. 14, 136–142. 10.1159/000526393 37064336 PMC10090967

[B11] Dorum S. Havalı C. Görükmez Görükmez O. (2020). Two patients with glutaric aciduria type 3: a novel mutation and brain magnetic resonance imaging findings. Turk J. Pediatr. 62, 657–662. 10.24953/turkjped.2020.04.017 32779420

[B12] Eggerding F. A. Schonberg S. A. Chehab F. F. Norton M. E. Cox V. A. Epstein C. J. (1994). Uniparental isodisomy for paternal 7p and maternal 7q in a child with growth retardation. Am. J. Hum. Genet. 55, 253–265. 7913578 PMC1918369

[B13] Eggermann T. (2010). Russell-Silver syndrome. Am. J. Med. Genet. C Semin. Med. Genet. 154c, 355–364. 10.1002/ajmg.c.30274 20803658

[B14] Fares F. David M. Lerner A. Diukman R. Lerer I. Abeliovich D. (2006). Paternal isodisomy of chromosome 7 with cystic fibrosis and overgrowth. Am. J. Med. Genet. A 140, 1785–1788. 10.1002/ajmg.a.31380 16835920

[B15] Feuk L. Kalervo A. Lipsanen-Nyman M. Skaug J. Nakabayashi K. Finucane B. (2006). Absence of a paternally inherited FOXP2 gene in developmental verbal dyspraxia. Am. J. Hum. Genet. 79, 965–972. 10.1086/508902 17033973 PMC1698557

[B16] Goh D. L. Zhou Y. Chong S. S. Ngiam N. S. Goh D. Y. (2007). Novel CFTR gene mutation in a patient with CBAVD. J. Cyst. Fibros. 6, 423–425. 10.1016/j.jcf.2007.02.004 17398169

[B17] Höglund P. Holmberg C. De La Chapelle A. Kere J. (1994). Paternal isodisomy for chromosome 7 is compatible with normal growth and development in a patient with congenital chloride diarrhea. Am. J. Hum. Genet. 55, 747–752. 7942853 PMC1918292

[B18] Joshi R. S. Garg P. Zaitlen N. Lappalainen T. Watson C. T. Azam N. (2016). DNA methylation profiling of uniparental disomy subjects provides a map of parental epigenetic bias in the human genome. Am. J. Hum. Genet. 99, 555–566. 10.1016/j.ajhg.2016.06.032 27569549 PMC5011056

[B19] Kotzot D. (2008). Complex and segmental uniparental disomy updated. J. Med. Genet. 45, 545–556. 10.1136/jmg.2008.058016 18524837

[B20] Kotzot D. Holland H. Keller E. Froster U. G. (2001). Maternal isochromosome 7q and paternal isochromosome 7p in a boy with growth retardation. Am. J. Med. Genet. 102, 169–172. 10.1002/ajmg.1430 11477611

[B21] Le Caignec C. Isidor B. De Pontbriand U. David V. Audrezet M. P. Ferec C. (2007). Third case of paternal isodisomy for chromosome 7 with cystic fibrosis: a new patient presenting with normal growth. Am. J. Med. Genet. A 143a, 2696–2699. 10.1002/ajmg.a.31999 17935233

[B22] Liehr T. (2023). Cases with uniparental disomy. Available at: http://upd-tl.com/upd.html (Accessed August 1, 2023).

[B23] Liehr T. (2010). Cytogenetic contribution to uniparental disomy (UPD). Mol. Cytogenet 3, 8. 10.1186/1755-8166-3-8 20350319 PMC2853554

[B24] Liehr T. (2014). Uniparental disomy (UPD) in clinical genetics: A guide for clinicians and patients. Heidelberg: Springer. 10.1007/978-3-642-55288-5

[B25] Marlaire S. Van Schaftingen E. Veiga-Da-Cunha M. (2014). C7orf10 encodes succinate-hydroxymethylglutarate CoA-transferase, the enzyme that converts glutarate to glutaryl-CoA. J. Inherit. Metab. Dis. 37, 13–19. 10.1007/s10545-013-9632-0 23893049

[B26] Nakamura A. Muroya K. Ogata-Kawata H. Nakabayashi K. Matsubara K. Ogata T. (2018). A case of paternal uniparental isodisomy for chromosome 7 associated with overgrowth. J. Med. Genet. 55, 567–570. 10.1136/jmedgenet-2017-104986 29455159

[B27] Nakka P. Pattillo Smith S. O'donnell-Luria A. H. Mcmanus K. F. Mountain J. L. Ramachandran S. (2019). Characterization of prevalence and Health consequences of uniparental disomy in four million individuals from the general population. Am. J. Hum. Genet. 105, 921–932. 10.1016/j.ajhg.2019.09.016 31607426 PMC6848996

[B28] Niska-Blakie J. Gopinathan L. Low K. N. Kien Y. L. Goh C. M. F. Caldez M. J. (2020). Knockout of the non-essential gene SUGCT creates diet-linked, age-related microbiome disbalance with a diabetes-like metabolic syndrome phenotype. Cell. Mol. Life Sci. 77, 3423–3439. 10.1007/s00018-019-03359-z 31722069 PMC7426296

[B29] Pan Y. Mccaskill C. D. Thompson K. H. Hicks J. Casey B. Shaffer L. G. (1998). Paternal isodisomy of chromosome 7 associated with complete situs inversus and immotile cilia. Am. J. Hum. Genet. 62, 1551–1555. 10.1086/301857 9585585 PMC1377136

[B30] Papenhausen P. Schwartz S. Risheg H. Keitges E. Gadi I. Burnside R. D. (2011). UPD detection using homozygosity profiling with a SNP genotyping microarray. Am. J. Med. Genet. A 155a, 757–768. 10.1002/ajmg.a.33939 21594998

[B31] Prawitt D. Haaf T. (2020). Basics and disturbances of genomic imprinting. Med. Genet. 32, 297–304. 10.1515/medgen-2020-2042

[B32] Reik W. Walter J. (2001). Genomic imprinting: parental influence on the genome. Nat. Rev. Genet. 2, 21–32. 10.1038/35047554 11253064

[B33] Saal H. M. Harbison M. D. Netchine I. (1993). “Silver-russell syndrome,” in GeneReviews(®). Editors Adam M. P. Mirzaa G. M. Pagon R. A. Wallace S. E. Bean L. J. H. Gripp K. W. (Seattle (WA): University of Washington, Seattle). 20301499

[B34] Scuffins J. Keller-Ramey J. Dyer L. Douglas G. Torene R. Gainullin V. (2021). Uniparental disomy in a population of 32,067 clinical exome trios. Genet. Med. 23, 1101–1107. 10.1038/s41436-020-01092-8 33495530 PMC8187148

[B35] Simpson M. A. Hsu R. Keir L. S. Hao J. Sivapalan G. Ernst L. M. (2007). Mutations in FAM20C are associated with lethal osteosclerotic bone dysplasia (Raine syndrome), highlighting a crucial molecule in bone development. Am. J. Hum. Genet. 81, 906–912. 10.1086/522240 17924334 PMC2265657

[B36] Waters P. J. Kitzler T. M. Feigenbaum A. Geraghty M. T. Al-Dirbashi O. Bherer P. (2018). Glutaric aciduria type 3: three unrelated Canadian cases, with different routes of ascertainment. JIMD Rep. 39, 89–96. 10.1007/8904_2017_49 28766179 PMC5953897

[B37] Yauy K. De Leeuw N. Yntema H. G. Pfundt R. Gilissen C. (2020). Accurate detection of clinically relevant uniparental disomy from exome sequencing data. Genet. Med. 22, 803–808. 10.1038/s41436-019-0704-x 31767986 PMC7118024

