# Supplementary material for: Case report: Paternal uniparental disomy on chromosome 7 and homozygous SUGCT mutation in a fetus with overweight after birth
Source: Front Genet. 2023 Oct 17;14:1272028. doi: 10.3389/fgene.2023.1272028 (PMC10619901; doi:10.3389/fgene.2023.1272028)
Supplement: Supplementary file 1 [file Table1.DOC]

**TABLE 1** Summary of cases with paternal UPD(7)

| **Case number** | **Gender** | **Age of UPD diagnosis** | **Karyotype** | **Other genetic abnormalities** | **Clinical symptoms** | **Growth** |
| --- | --- | --- | --- | --- | --- | --- |
| **Case 1** | F | 6 mo | 46,XX,i(7)(p10),i(7)(q10) | maternal isoUPD(7q) and paternal isoUPD(7p) | growth retardation, SRS like | growth-restricted |
| **Case 2** | M | 12 y | 46,XY,i(7)(p10),i(7)(q10) | maternal isoUPD(7q) and paternal isoUPD(7p) | growth retardation, SRS like | growth-restricted |
| **Case 3** | M | NB | 45,XY,psu dic(7;7)(p22;p22) | 7p telomeric microdeletion and mutation of *FAM20C*in 7p22.3 | lethal osteosclerotic bone dysplasia (died 2 h after birth) | NA |
| **Case 4、Case 5** | NA | NA | NA | NA | NA | NA |
| **Case 6** | M | 6 mo | N | mutation of *CFTR* in 7q31.2 and *DNAH11* in 7p21 | CF, primary ciliary dyskinesia, situs inversus | N |
| **Case 7** | F | 3 y | N | mutation of *CFTR* in 7q31.2 | CF | N |
| **Case 8** (case 17) | M | postnatal | N | mutation of *CFTR* in 7q31.2 | CF, mild language delay | N |
| **Case 9** | M | 33 y | N | mutation of *CFTR* in 7q31.2 | CF, congenital bilateral absence of vas deferens | N |
| **Case 10** | M | 2 y | N | mutation of *CFTR* in 7q31.2 | CF, delayed development, overweight | overweight |
| **Case 11** | F | 23 y | N | mutation of *SLC26A3* in 7q31.1 | congenital chloride diarrhea, sensorineural hearing loss | N |
| **Case 12** | M | 5 y | N | no disease causing gene identified | overgrowth, mitral regurgitation | overgrowth |
| **Case 13** | M | NB | N | UPD(15)pat and UPD(7)pat | Beckwith–Wiedemann syndrome spectrum | overgrowth |
| **Present case** | M | prenatal | N | mutation of *SUGCT* in 7p14.1 | overweight | overweight |

M, male; F, female; y, years; mo, months; NB, newborn; NA, not available; N, normal; isoUPD, isodisomy; CF, cystic fibrosis; SRS, Silver-Russell syndrome.
